# Perforated Acute Appendicitis in a Pre-Term Neonate

**DOI:** 10.5812/ircmj.4629

**Published:** 2013-06-05

**Authors:** Mohammad Jahangiri, Mehrdad Hosseinpour, Hoda Jazayeri, Mahdi Mohammadzadeh, Davood Motaharizad, Azadeh Sadat Mirzadeh

**Affiliations:** 1 Trauma Research Centre, Kashan University of Medical Sciences, Kashan, IR Iran

**Keywords:** Infant, Newborn, Appendicitis, Peritonitis

## Abstract

Acute appendicitis is a common occurrence in childhood, but this diagnosis is considered rarely in differential diagnosis of acute abdomen in the neonatal period because its occurrence is very rare in neonates. We report a 20-day- old afghan female baby that was admitted to neonatal intensive care unit, because of irritability and abdominal distension. Complete ultrasound of abdomen and pelvis was normal. In plain Radiographs of chest and abdomen with the exception of Air-filled stomach and intestine, there was no abnormality. Due to the lack of improvement and severe abdominal distension, she was transmitted to the operating room and Surgical exploration revealed perforated appendix. Appendicitis should be considered in the differential diagnosis for a neonate with abdominal distension and bilious vomiting and needs strong clinical suspicion.

## 1. Introduction

Although appendicitis is a common disease in children and young adult, but its occurrence is very rare in neonates.Appendicitis during neonatal period, especially in perforated cases has high mortality and morbidity. Only few cases are reported in literature and only three cases have been reported in Indian literature (1-3). In this report, we discussed a case of neonatal appendicitis with perforations, presented with features of generalized peritonitis.

## 2. Case Report

The patient was a 20-day- old afghan female baby that was admitted to neonatal intensive care unit (NICU), because of irritability and abdominal distension .She was pre-term with gestational age of thirty two weeks, born by cesarean section (C / S) due to placental abruption and placenta previa. Her birth weight was1900 grams (gr) and had a history of ten days staying in NICU. The patient's symptoms began the day before admission and gradually increased. During this time she had two episodes of non-bilious vomiting, associated with normal defecation and no fever.

In physical examination she was irritable and had prominent abdominal distension and icteric skin. In respiratory examination, she had mild respiratory distress and inter-costal retraction with respiratory rate about 60/min. Rectal and other examinations were normal.She was admitted to NICU with primary diagnosis of partial bowel obstruction or necrotizing enterocolitis.

**Figure 1.**
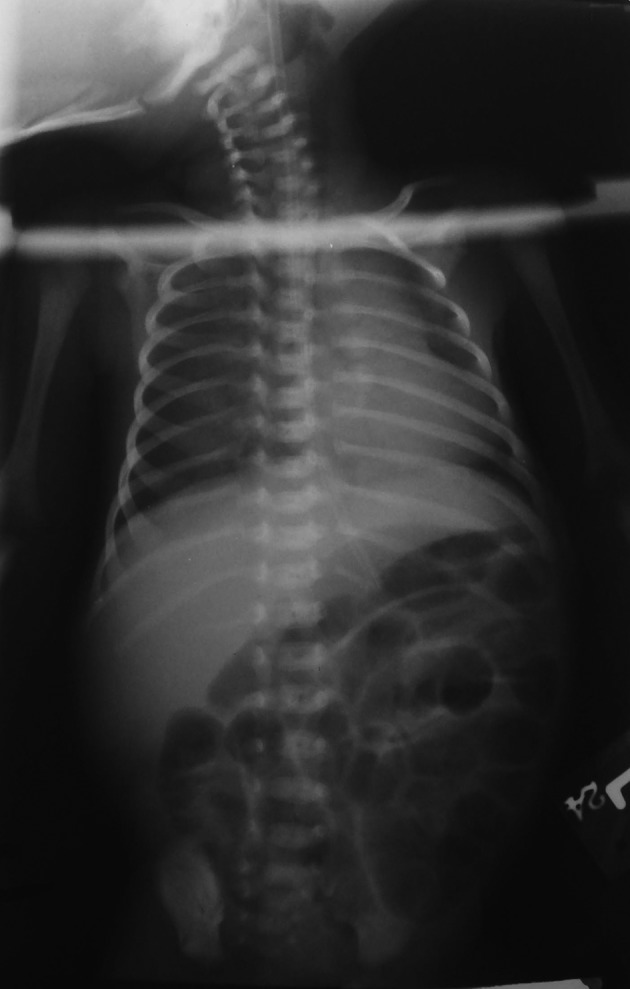
Chest and abdomen plain Radiograph

In laboratory tests, the WBC count was 13700/mm ^3 ^with PMN 62.5%, hemoglobin; 7.5 g/dL, HCT; 23.6%, platelet count; 5.5×106 /mm ^3 ^, total bilirubin; 14.5mg/dl with Direct bilirubin of 0.9mg/dl ,retic count; 2%,S/E:OB1+ and CRP; negative. Laboratory tests for liver and kidney were normal. Complete ultrasound of abdomen and pelvis was normal. In plain Radiographs of chest and abdomen with the exception of Air-filled stomach and intestine,there was no abnormality (Figure1).

She was stabilized with appropriate fluid management, kept nil per orally and NGT Tube was placed and surgical consultation requested. Antibiotic therapy consisting of cefotaxime, vancomycin, and metronidazole was administered.Two unit pack cells were infused because of anemia and the latest hemoglobin was 13mg/dl. Phototherapy for jaundice was performad and the latest bilirubin was 6.7mg/dl.Due to the lack of improvement, severe abdominal distension and also one episode of bilious vomiting, she was transmitted to the operating room for exploratory laparotomy. Surgical exploration revealed perforated appendix and peritonitis combined with purulent abdominal discharge, Inter loop abscess and grossly perforated appendix, so the abscess was drained and appendectomy was done in classical method.

Histopathologic evaluation of the resection specimen showed an appendix 3 cm in length, with neutrophilic exudate in the lumen, muscular layer and surrounding fat. This confirmed the diagnosis of acute suppurative appendicitis with peri-appendicitis. Post-operative period was uneventful. Feeding initiated on day 5 by gavage and then gradually was switched to breast feeding. The baby discharged from the NICU with good health.

## 3. Discussion

Acute appendicitis is a common occurrence in childhood, but this diagnosis is considered rarely in differential diagnosis of acute abdomen in the neonatal period. The incidence of appendicitis in neonate varies from 0.04 to 0.2% and is more common in premature neonate (4-6), mortality rate in neonatal appendicitis was 78% in the 1901–1975 period and has decreased to 33% in the 1976–1984 period, and to 28% in the1985–2000 period (7). The low incidence of acute appendicitis during infancy is due to several factors including the embryonic form of appendix (funnel shape with wide entry into the cecum) so the appendix is less prone to be walled-off exactly opposite its finger-like form in older children (2, 7, 8). Intraluminal obstruction is unlikely because of the curved posture of the appendix and also the liquid diet (8). Evaluation of the symptoms of appendicitis in the neonatal period is extremely difficult which eventually leads to delayed diagnosis, resulting in an increased rate of perforation, peritonitis and mortality (2, 8).

Clinical signs of acute appendicitis iclude irritability, respiratory distress and wriggling that confirm peritoneal inflammation. This also may be associated with swelling of the scrotum and a right lower quadrant mass. Patients lesley present anorexia, fever and leukocytosis (7, 8). Abdominal radiograph may show abnormal gas patterns, air fluid level, right scoliosis and disappearance of Psoas margin.Calcified appendicolith is seen in some of older patients's X-ray that has not been reported in neonatal period (7, 8). Spiral computed tomography can be useful as diagnostic tools as well but (9), there is concern about the potential radiation-related cancer risk (10). Perforation is an important factor in determining of prognosis, because of having no specific symptoms, the incidence of perforations in the neonatal period is very high. Other reasons are a thin appendiceal wall and a non-dilated cecum (11). A relatively small size and under developed peritoneal space with lower physiologic reserve are possible explanations for rapid spread of infection. These factors may contribute to high neonatal mortality rate due to perforation and peritonitis (12). The perforated appendicitis in this age group can be due to Hirschsprung's disease, meconium plug syndrome, cystic fibrosis, necrotizing enterocolitis and gastroenteritis (13). So it is crucial to histological diagnosis and assessment of sample was taken, if it is possible, samples of colon and rectum should be taken (13, 14).

## 4. Conclusion

Neonatal appendicitis continues to be a diagnostic challenge and strong clinical suspicion. Appendicitis should be considered in the differential diagnosis for a neonate with abdominal distension and bilious vomiting and needs for aggressive investigations such as laparotomy must be considered to improve outcomes.
